# Lysogenic Conversion and Phage Resistance Development in Phage Exposed *Escherichia coli* Biofilms

**DOI:** 10.3390/v5010150

**Published:** 2013-01-11

**Authors:** Pieter Moons, David Faster, Abram Aertsen

**Affiliations:** Laboratory of Food Microbiology, Department of Microbial and Molecular Systems (M^2^S), Faculty of Bioscience Engineering, Katholieke Universiteit Leuven, Kasteelpark Arenberg 22, 3001 Leuven, Belgium; E-Mails: pieter.moons@ua.ac.be (P.M.); david.faster@biw.kuleuven.be (D.F.)

**Keywords:** *Escherichia coli*, biofilm, lysogenic conversion, resistance development, Shiga toxin, phage

## Abstract

In this study, three-day old mature biofilms of *Escherichia coli* were exposed once to either a temperate Shiga-toxin encoding phage (H-19B) or an obligatory lytic phage (T7), after which further dynamics in the biofilm were monitored. As such, it was found that a single dose of H-19B could rapidly lead to a near complete lysogenization of the biofilm, with a subsequent continuous release of infectious H-19B particles. On the other hand, a single dose of T7 rapidly led to resistance development in the biofilm population. Together, our data indicates a profound impact of phages on the dynamics within structured bacterial populations.

## 1. Introduction

Many bacterial populations are organized as biofilms, which consist of cells attached to a surface embedded in a matrix of variable composition [1]. These biofilms structurally vary from flat layers of cells to complex structured communities, consisting of tower or mushroom shaped micro-colonies interspersed with water channels that allow access of nutrients and removal of metabolites [2,3,4]. Bacterial biofilms present a medical hazard, since they confer increased resistance against antimicrobials and the host immune system [5], and are linked to persistent infections [6,7,8]. 

Apart from being a predominant species among the facultative anaerobic bacteria in the gastrointestinal tract [9], *E. coli* is considered a major zoonotic food-borne pathogen [10] and is one of the main causes of nosocomial infections [11], such as those associated with biofilm formation on urinary catheters [12]. Based on their disease-associated virulence factors, the species is divided into pathotypes, which are known to cause diarrhoeal disease, extra-intestinal and urinary tract infections, sepsis and meningitis [13,14]. Virulence characteristics include a variety of adhesion and colonization factors, the formation of attaching and effacing lesions, the production of toxins and the presence of antibiotic resistance genes [13,15,16]. Among diarrheagenic *E. coli*, those producing the potent Shiga (or Vero) toxins (Stxs) are the most virulent and can cause the potentially fatal haemolytic uremic syndrome [17,18]. Genes encoding Stxs are typically harbored by temperate phages (Stx-phages) integrated in the genome of such pathogenic *E. coli* strains, which makes them subject to lateral gene transfer [19,20].

The worldwide emergence of multi-drug resistant pathogens and empty antibiotic development pipelines reduce medical treatment options and necessitate the research into alternative therapies. One such option is phage therapy, which makes use of lytic phages as natural bacterial enemies whose narrow host range minimizes their impact on the normal flora [21,22,23,24]. In fact, phages are well suited to affect biofilms, since infection leads to local enrichment of viral particles. Moreover, many bacteriophages possess enzymes capable of bacterial lysis and less common biofilm matrix degradation [22,25,26], aiding in the accessibility of biofilm cells toward viral particles [27,28] and leading to biofilm dispersal [29,30,31]. 

In this study, we examined the impact of a temperate or lytic phage on mature *E. coli* biofilms, with particular interest in the lateral transfer of virulence determinants and phage resistance development, respectively. 

## 2. Results and Discussion

### 2.1. Lysogenic Conversion of a Mature E. coli Biofilm with an Stx-Encoding Phage

In order to examine to which extent phage encoded virulence factors could be captured and spread within an existing *E. coli *biofilm, we decided to make acquisition of the naturally *stx1*-encoding temperate H-19B phage [32] readily detectable through selective plating by equipping it with an antibiotic resistance marker. Since only a small fragment of the H-19B genome sequence is currently known [32], a random transposon mutagenesis procedure was followed to tag this (pro)phage. More specifically, *E. coli* MG1655 was first lysogenized with H-19B, after which a random Tn10-transposon library of ca. 10,000 clones was constructed in this lysogen using the λNK1324 hop protocol described by Kleckner *et al.* [33]. Assuming some of the Tn10 insertions to be located within the H-19B prophage, mitomycin C was subsequently used to induce the prophage in the obtained pool of transposon mutants. To isolate H-19B::Tn10 mutants within the corresponding phage lysate, it was first plaqued on *E. coli* MG1655. Since the Tn10 transposon codes for the *cat* gene and confers chloramphenicol resistance, lysogens arising in the middle of turbid plaques were scored for the presence or absence of chloramphenicol resistance. As such, nine chloramphenicol resistant lysogens were obtained, which were further confirmed to simultaneously have acquired the *stx1* operon of H-19B. The main advantage of this procedure is that it automatically disregards phage mutants compromised in lytic or lysogenic development. Moreover, in contrast to phage recombineering protocols [34], this protocol can be applied without prior knowledge of the phage’s genome sequence and resembles the method used previously by Acheson *et al.* [35] to look for phage encoded exported proteins.

**Figure 1:**
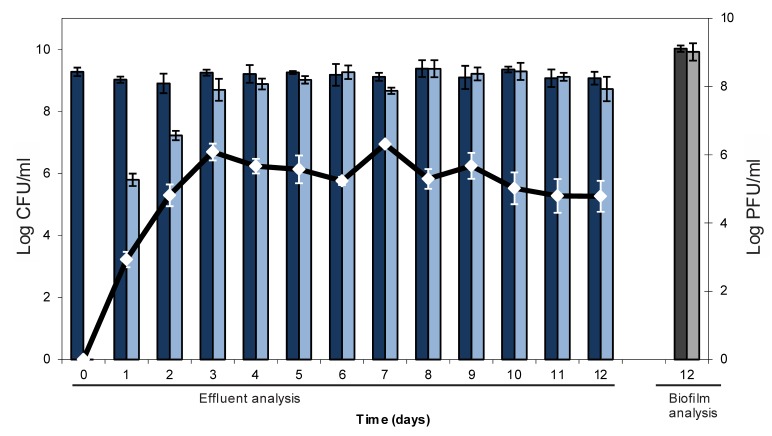
Numbers of viable cells (CFU/mL) and phages (PFU/mL) in the effluent of a mature *E. coli* biofilm exposed for 1 hour to 50 PFU/mL (total of 150 PFU per biofilm) of H-19B::Tn10 phage at day 0, after sampling the effluent for initial total cell count. Dark blue bars represent total cell concentration, while light blue bars represent the concentration of H-19B::Tn10l ysogens. White markers represent free H-19B::Tn10 phage. Dark and light grey bars represent total cell counts and lysogen cell counts, respectively, in the attached biofilm obtained after dismantling the flow cell setup. The data represent the average and standard deviations of three biological replicates.

From one of the obtained H-19B::Tn10 lysogens, the Tn10 insertion site was determined and found to map within a gene bearing homology to the *nleG* virulence genes. NleG proteins are effectors of the type 3 secretion system that are thought to mimic eukaryotic E3 ubiquitin ligases [36], and are generally found in the late region of phage genomes [37]. This insertion underscores the presence of additional virulence genes to be present in H-19B, supports the observation that neither lytic nor lysogenic behaviour is affected in H-19B::Tn10 and due to the uptake of an additional piece of DNA (*i.e*,*.* the transposon) adds evidence to the plasticity of Stx genomes [38]. Subsequently, a mature three-day old *E. coli* biofilm was only once exposed to a small number (*i.e.*, 150 viral particles spread over one hour) of the corresponding H-19B::Tn10 derivative in order to mimic an accidental exposure. Subsequently, the spread of this virulence conferring phage genome throughout the biofilm population was tracked. More specifically, the effluent of the biofilm was examined for the presence of (i) cells lysogenized with H-19B::Tn10, and (ii) free H-19B::Tn10 phage (Figure 1). Interestingly, from this analysis it became clear that lysogenic conversion of the biofilm proceeded very rapidly, with the emergence of *ca.* 10^6^ CFU/mL of H-19B::Tn10 lysogens on a total effluent cell count of circa 10^9^ CFU/mL (*i.e.*, 0.06% conversion) after 24 h, and an above 50% conversion reached after five days. Moreover, at the end of the experiment the ratio between total and lysogenized cells within the actual biofilm itself corresponded to that observed in the effluent, demonstrating that, at least at the end of the experiment, lysogenic conversion had occurred in the entire biofilm Furthermore, during the first two days after phage exposure, the concentration of free H-19B::Tn10 phage in the effluent quickly rose from the applied 50 PFU/mL to 10^5^–10^6^ PFU/mL, after which it remained stable at this level. It can be anticipated that part of the biofilm is being lysed, leading to a local enrichment in H-19B::Tn10 concentration. Nevertheless, it remains unclear exactly to what extent the emergence of H-19B::Tn10 lysogens is the result of *de novo* lysogenic conversion of pre-existing wild-type cells or of clonal enrichment of the first converted cells.

### 2.2. Resistance Development of E. coli biofilms Against a Lytic Phage

**Figure 2:**
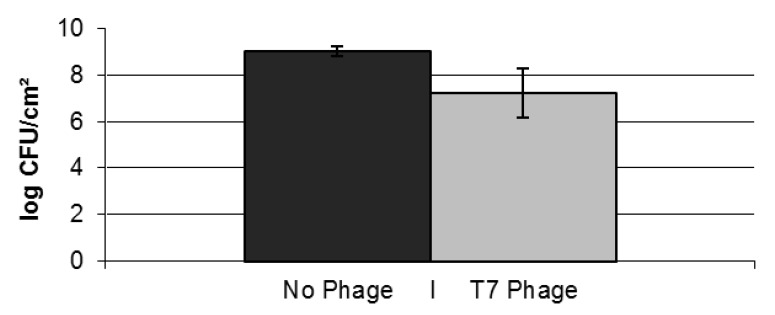
Number of viable cells (CFU) within a mature 3-day old *E. coli* biofilm exposed once, for one hour, to either water (control; dark bars) or *ca.* 10^8^ PFU/mL of T7 phage (light bars) followed by an additional three days of biofilm development. The data represent the average and standard deviations of three biological replicates.

**Figure 3:**
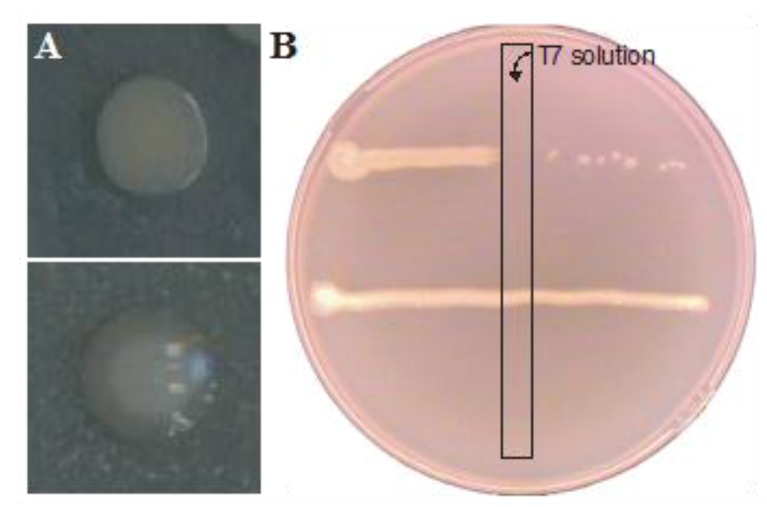
(**A**) mucoid (bottom) and non mucoid (top) colony of *E. coli*. (**B**) Phage resistance of a bacterial colony was demonstrated by streaking a line of *E. coli* cells through a perpendicularly streaked line of T7 solution. T7 phage resistant cells (bottom) were capable of growth past this line, while T7 sensitive cells (top) were not.

In a second approach, biofilm dynamics were examined upon single exposure of a 3-day old mature *E. coli* biofilm to *ca.* 10^8^ particles per milliliter of the obligatory lytic T7 phage. The high phage titer selected in this approach was considered to best reflect the clinical application of phage in combatting biofilm-related infections. After three additional days of incubation, cells within the biofilm were harvested and enumerated. A comparison between the number of viable cells within the T7-exposed or -unexposed biofilm revealed an almost 100-fold reduction (*p*-value in two tailed homoscedastic T-test <0.05) in the phage exposed biofilm (Figure 2). However, in between the normal colonies observed during plating, colonies with a mucoid appearance were observed in biofilms treated with T7 phages (Figure 3A), but not in untreated biofilms. This phenotype was earlier shown to be correlated with T7 resistance due to a mutation resulting in the production of excess capsular polysaccharide preventing T7 adhesion [39] due to physical blocking of the phage binding site [40], and actual T7 resistance of these colonies could be confirmed as in Figure 3B. After examining *ca*. 400 colonies of both types, phage resistant clones with a non-mucoid phenotype or phage sensitive clones with a mucoid phenotype were not observed. In turn, this enabled us to accurately determine the number of resistant cells in the biofilm. However, a large variation in the number of resistant cells was found between biological replicates (Table 1), ranging from circa 0.05% to over 28%. As in Luria-Delbruck fluctuation experiments, this variation likely stemmed from clonal enrichment of stochastically pre-existing T7-resistant mutants that spontaneously arise within a population. Since 3 days after initial phage exposure T7 particles still remained present in the effluent and the biofilm, on-going selection for T7-resistance development seems warranted upon longer incubation of the biofilm. 

**Table 1:** Viable cell counts within three mature three-day old *E. coli* biofilms each exposed once for one hour to *ca.* 10^8^ PFU/mL of T7 phage, followed by three additional days of growth before plating.

Cell counts (log CFU/cm²)	Biofilm 1	Biofilm 2	Biofilm 3
Total cell count	7.31	5.82	7.91
T7 resistant cells	6.76	2.49	7.05
T7 sensitive cells	7.17	5.82	7.85

## 3. Experimental Section

### 3.1. Strains, Standard Culture Conditions and Chemicals

*E. coli* MG1655 [41], MG1655 *lacZ::Tc *[42], and MG1655 H-19B (*i.e.*, lysogenized with *stx1* encoding phage H-19B; [43]) were used in this study, and were cultured at 37 °C under shaking conditions in Lysogeny Broth (LB). In order to determine the number of bacteria (as colony forming units or CFU) in a sample, a dilution series was prepared and plated on LB agar (1.5% agar), after which the concentration was expressed as CFU/mL.

To obtain a H-19B phage lysate from lysogens, cells from 1 mL bacterial culture were pelleted by centrifugation (6000 × g, 15 min), after which 50 µL of CHCl_3_ was added to sterilize the supernatant and to release progeny phage from infected but non-lysed cells. In case high phage titers were required, the cultures were grown in the presence of 2 µg/mL mitomycin C which induces the release of temperate phages. To obtain a T7 phage [44] lysate, T7 was propagated on MG1655, after which 1 mL of cells were pelleted by centrifugation (6000 × g, 15 min) and the resulting supernatant was sterilized by addition of 50 µL of CHCl_3_. In order to determine the number of phages (as plaque forming units or PFU) in a sample of lysate or effluent, a dilution series was prepared and plated on *E. coli* MG1655 grown in LB soft agar (0.7% agar), after which the concentration was expressed as PFU/mL.

Where necessary, growth media were supplemented with tetracycline (20 µg/mL) and/or chloramphenicol (30 µg/mL) (Applichem, Darmstadt, Germany).

### 3.2. Analysis of H-19B::Tn10

After its construction as described in the text, the Tn10 insertions site in H-19B::Tn10 was mapped by subcloning of the Tn10 encoded chloramphenicol resistance marker in pUC18. Subsequently, genomic DNA flanking the Tn10 transposon was sequenced using a primer (5’-AAGCACCGCCGGACATC-3’) reading outwards of the transposon. In addition, the isolated H-19B::Tn10 phage was also confirmed by PCR (directly on plaques or crude lysate) to still carry its stx1 locus, by using primers flanking this region (5’-CAGTGGATCCTGGCACGGAAACATGGGT-3’ and 5’-TCAGTCTAGATTACGTCTTTGCAGT CGAGAAGTC-3’).

### 3.3. Setup for Biofilm Formation

Biofilms were grown at 30 ºC in three-channel flow chambers (Biocentrum DTU: Technical University of Denmark, Soltofts Plads 221 DK-2800 Kgs, Lyngby) with individual channel dimensions of 1 × 4 × 40 mm^3^ [45] that were covered with a microscope glass coverslip (st1; Knittel Gläser, Braunschweig, Germany). The setup makes use of a 16-channel peristaltic pump (Watson Marlow 205S, Zellik, Belgium) that feeds each channel with a flow of 3 mL/h (flow rate of 0.2 mm/s) of AB-trace medium [2 g/L (NH_4_)_2_SO_4_; 6 g/L Na_2_HPO_4_.2H_2_0; 3 g/L KH_2_PO_4_; 3 g/L NaCl; 9.5 mg/L MgCl_2_; 11.1mg/L CaCl_2_ and 0.1 mL/L of the following trace metal mixture (200 mg/L CaSO_4_.2H_2_O; 200 mg/L FeSO_4_.7H_2_O; 20 mg/L MnSO_4_.H_2_O; 20 mg/L CuSO_4_.5H_2_O; 20 mg/L ZnSO_4_.7H_2_O; 10 mg/L CoSO_4_.7H_2_O; 10 mg/L NaMoO_4_.H_2_O; 5 mg/L H_3_BO_3_)] supplemented with 0.3 mM glucose and 1 µg/mL thiamine dichloride. Bubble traps were placed in each channel before the flow cell to remove air bubbles. Before use, the flow system was sterilized by flushing with a solution of 0.5% sodium hypochlorite for 4 h, and rinsed with approximately 0.2 L of sterile water before the medium was pumped through. 

Bacterial cultures for inoculation were prepared by diluting an overnight LB broth culture 1/100 in fresh LB medium and regrowing it for 4 h at 30 ºC under shaking conditions. To inoculate the flow cells the medium flow was stopped, flow chambers were turned with the glass coverslip down and 250 µL of the diluted cell suspension was carefully injected through the silicon tubes into each flow channel with a small syringe. After 1 h, to allow adsorption of the cells to the coverslip surface, the flow channels were turned upright and the flow was resumed. For experiments with H-19B::Tn10 and T7, *E. coli* MG1655 *lac::Tc* and *E. coli* MG1655 were used, respectively. When macroscopically visible mature *E. coli* biofilms were formed after 3 days, they became exposed for 1 h to either sterile water, 50 PFU/mL of H-19B::Tn10 or *ca.* 10^8^ PFU/mL of T7 via a separate input channel. 

### 3.4. Analysis of Biofilms

Biofilms were analyzed by determining the number of viable cells (CFU) or phage particles (PFU) in either the effluent or in the biofilm itself. Effluent was collected using a small connector inserted in the tubing behind the flowcell, while the actual biofilm was harvested by carefully disconnecting the flowcells and vigorously pipetting up and down the channels with 250 µL potassium phosphate buffer (10 mM, pH 7.0). 

After vortexing, dilution series of collected samples were plated on LB agar (in case *E. coli* MG1655 was used) or LB agar supplemented with tetracycline (in case *E. coli* MG1655 *lac::Tc* was used) to enumerate total cell counts, and on LB agar supplemented with chloramphenicol to enumerate H-19B::Tn10 lysogens. Phages were enumerated by first sterilizing the collected samples with CHCl_3_, and subsequently plating them on *E. coli* MG1655.

## 4. Conclusions

Biofilms, already recalcitrant to therapy, can become an even larger burden through the acquisition of novel virulence determinants such as those conveyed by temperate phages. In Stx phages, the toxin genes are associated with the late gene region, and become highly expressed when the (pro)phage enters the lytic cycle which can be induced at initiation of the bacterial SOS response [19,20,46]. Of intestine isolated *E. coli* strains, 10–30% were shown to be able to contribute to Shiga toxin production upon infection through transfer of the phage [19,47,48], while also actual *in vivo* transfer was demonstrated [35,49,50]. Certain antibiotics, through induction of the SOS response [51], even lead to the release of temperate phages [52] and thus contribute to the spread of phages (and genes) to the normal flora and elevated production of toxins.

In this study, we report the integration of a chloramphenicol resistance marker in the temperate H-19B Stx-phage without compromising either the lysogenic or the lytic cycle. This phage was shown to rapidly and massively establish itself as a prophage within a mature *E. coli *biofilm, despite the very low initial dose. During and after this establishment, the biofilm started to produce a high number of free H-19B virions, thus contributing to the further dissemination of the phage and its virulence determinants. Moreover, since the production and release of H-19B phage typically coincides with the production and release of its Shiga-toxin [19,20,53], such biofilms likely become a source of continuous toxin production. These findings underscore the ability of temperate phages to rapidly and stably establish themselves within a susceptible but matrix-embedded population, and demonstrate that biofilms can serve as a source of new phage particles and their possible toxins. 

Upon challenging a biofilm with lytic phages, an initial reduction in cell numbers is often observed [54,55], but complete eradication by a single phage is never achieved and the establishment of equilibrium between virus and host with stable numbers of both organisms was reported earlier [56]. In addition, resistance is thought to quickly arise within the biofilm, but is only scarcely supported by quantitative data. Here, we report the application of a single pulse of lytic T7 phage to an existing *E. coli *biofilm and demonstrate a reduction of the biofilm even several days after phage application. Nevertheless, this lysis was far from complete and, although highly variable, up to 30% of phage resistant cells could be isolated. This development of resistance was earlier described as a mutual, escalating arms race in which phage arise that are capable of infecting resistant strains, from which in turn bacteria evolve that are resistant to those phages [57,58]. These results confirm the therapeutic potential of lytic phages mainly lies in the use of either well-characterized phage cocktails or by combining them with antibiotics. Synergistic phage-antibiotic combinations on *E. coli *biofilms were observed earlier [59,60] and show great promise for adequate treatment. 

## References

[1] Flemming H.C., Wingender J. (2010). The biofilm matrix. Nat. Rev. Microbiol..

[2] Hall-Stoodley L., Costerton J.W., Stoodley P. (2004). Bacterial biofilms: from the natural environment to infectious diseases. Nat. Rev. Microbiol..

[3] Moons P., Michiels C.W., Aertsen A. (2009). Bacterial interactions in biofilms. Crit. Rev. Microbiol..

[4] Høiby N., Ciofu O., Johansen H.K., Song Z.J., Moser C., Jensen P., Molin S., Givskov M., Tolker-Nielsen T., Bjarnsholt T. (2011). The clinical impact of bacterial biofilms. Int. J. Oral Sci..

[5] Høiby N., Bjarnsholt T., Givskov M., Molin S., Ciofu O. (2010). Antibiotic resistance of bacterial biofilms. Int. J. Antimicrob. Agents.

[6] Costerton J.W., Stewart P.S., Greenberg E.P. (1999). Bacterial biofilms: a common cause of persistent infections. Science.

[7] Francolini I., Donelli G. (2010). Prevention and control of biofilm-based medical-device-related infections. FEMS Immunol. Med. Microbiol..

[8] Hall-Stoodley L., Stoodley P., Kathju S., Høiby N., Moser C., Costerton J.W., Moter A., Bjarnsholt T. (2012). Towards diagnostic guidelines for biofilm-associated infections. FEMS Immunol. Med. Microbiol..

[9] Beloin C., Roux A., Ghigo J.M. (2008). *Escherichia coli *biofilms. Curr. Top. Microbiol. Immunol..

[10] Newell D.G., Koopmans M., Verhoef L., Duizer E., Aidara-Kane A., Sprong H., Opsteegh M., Langelaar M., Threfall J., Scheutz F. (2010). Food-borne diseases—The challenges of 20 years ago still persist while new ones continue to emerge. Int. J. Food Microbiol..

[11] Cantón R., Akóva M., Carmeli Y., Giske C.G., Glupczynski Y., Gniadkowski M., Livermore D.M., Miriagou V., Naas T., Rossolini G.M. (2012). Rapid evolution and spread of carbapenemases among Enterobacteriaceae in Europe. Clin. Microbiol. Infect..

[12] Jacobsen S.M., Stickler D.J., Mobley H.L., Shirtliff M.E. (2008). Complicated catheter-associated urinary tract infections due to *Escherichia coli* and *Proteus mirabilis*. Clin. Microbiol. Rev..

[13] Kaper J.B., Nataro J.P., Mobley H.L. (2004). Pathogenic *Escherichia coli*. Nat. Rev. Microbiol..

[14] Chaudhuri R.R., Henderson I.R. (2012). The evolution of the *Escherichia coli* phylogeny. Infect. Genet. Evol..

[15] Johnson T.J., Nolan L.K. (2009). Pathogenomics of the virulence plasmids of *Escherichia coli*. Microbiol. Mol. Biol. Rev..

[16] Farfan M.J., Torres A.G. (2012). Molecular mechanisms that mediate colonization of Shiga toxin-producing *Escherichia coli* strains. Infect. Immun..

[17] Werber D., Krause G., Frank C., Fruth A., Flieger A., Mielke M., Schaade L., Stark K. (2012). Outbreaks of virulent diarrheagenic *Escherichia coli*—are we in control?. BMC Med..

[18] Melton-Celsa A., Mohawk K., Teel L., O'Brien A. (2012). Pathogenesis of Shiga-toxin producing *Escherichia coli*. Curr. Top. Microbiol. Immunol..

[19] Schmidt H. (2001). Shiga-toxin-converting bacteriophages. Res. Microbiol..

[20] Herold S., Karch H., Schmidt H. (2004). Shiga toxin-encoding bacteriophages—genomes in motion. Int. J. Med. Microbiol..

[21] Minot S., Sinha R., Chen J., Li H., Keilbaugh S.A., Wu G.D., Lewis J.D., Bushman F.D. (2011). The human gut virome: Inter-individual variation and dynamic response to diet. Genome Res..

[22] Donlan R.M. (2009). Preventing biofilms of clinically relevant organisms using bacteriophage. Trends Microbiol..

[23] Loc-Carrillo C., Abedon S.T. (2011). Pros and cons of phage therapy. Bacteriophage.

[24] Ryan E.M., Gorman S.P., Donnelly R.F., Gilmore B.F. (2011). Recent advances in bacteriophage therapy: how delivery routes, formulation, concentration and timing influence the success of phage therapy. J. Pharm. Pharmacol..

[25] Azeredo J., Sutherland I.W. (2008). The use of phages for the removal of infectious biofilms. Curr. Pharm. Biotechnol..

[26] Rodríguez-Rubio L., Martínez B., Donovan D.M., Rodríguez A., García P.  (2012). Bacteriophage virion-associated peptidoglycan hydrolases: potential new enzybiotics. Crit. Rev. Microbiol..

[27] Hughes K.A., Sutherland I.W., Jones M.V. (1998). Biofilm susceptibility to bacteriophage attack: The role of phage-borne polysaccharide depolymerase. Microbiology.

[28] Domenech M., García E., Moscoso M. (2011). *In vitro* destruction of *Streptococcus pneumoniae* biofilms with bacterial and phage peptidoglycan hydrolases. Antimicrob. Agents Chemother..

[29] Rice S.A., Tan C.H., Mikkelsen P.J., Kung V., Woo J., Tay M., Hauser A., McDougald D., Webb J.S., Kjelleberg S. (2009). The biofilm life cycle and virulence of *Pseudomonas aeruginosa* are dependent on a filamentous prophage. ISME J..

[30] Meng X., Shi Y., Ji W., Zhang J., Wang H., Lu C., Sun J., Yan Y. (2011). Application of a bacteriophagelysin to disrupt biofilms formed by the animal pathogen *Streptococcus suis*. Appl. Environ. Microbiol..

[31] Siringan P., Connerton P.L., Payne R.J., Connerton I.F. (2011). Bacteriophage-Mediated dispersal of *Campylobacter jejuni* biofilms. Appl. Environ. Microbiol..

[32] Neely M.N., Friedman D.I. (1998). Functional and genetic analysis of regulatory regions of coliphage H-19B: Location of shiga-like toxin and lysis genes suggest a role for phage functions in toxin release. Mol. Microbiol..

[33] Kleckner N., Bender J., Gottesman S. (1991). Uses of transposons with emphasis on Tn10. Meth. Enzymol..

[34] Serra-Moreno R., Acosta S., Hernalsteens J.P., Jofre J., Muniesa M. (2006). Use of the lambda Red recombinase system to produce recombinant prophages carrying antibiotic resistance genes. BMC Mol. Biol..

[35] Acheson D.W., Reidl J., Zhang X., Keusch G.T., Mekalanos J.J., Waldor M.K. (1998). *In vivo* transduction with shiga toxin 1-encoding phage. Infect. Immun..

[36] Wu B., Skarina T., Yee A., Jobin M.C., Dileo R., Semesi A., Fares C., Lemak A., Coombes B.K., Arrowsmith C.H. (2010). NleG Type 3 effectors from enterohaemorrhagic *Escherichia coli* are U-Box E3 ubiquitin ligases. PLoS Pathog..

[37] Ogura Y., Ooka T., Iguchi A., Toh H., Asadulghani M., Oshima K., Kodama T., Abe H., Nakayama K., Kurokawa K. (2009). Comparative genomics reveal the mechanism of the parallel evolution of O157 and non-O157 enterohemorrhagic *Escherichia coli*. Proc. Natl. Acad. Sci. USA.

[38] Smith D.L., Rooks D.J., Fogg P.C., Darby A.C., Thomson N.R., McCarthy A.J., Allison H.E. (2012). Comparative genomics of Shiga toxin encoding bacteriophages. BMC Genom..

[39] Radke K.L., Siegel E.C. (1971). Mutation preventing capsular polysaccharide synthesis in *Escherichia coli *K-12 and its effect on bacteriophage resistance. J. Bacteriol..

[40] Scholl D., Adhya S., Merril C. (2005). Escherichia coli K1's capsule is a barrier to bacteriophage T7. Appl. Environ. Microbiol..

[41] Guyer M.S., Reed R.R., Steitz J.A., Low K.B. (1981). Identification of a sex-factor-affinity site in *E. coli* as gamma delta. Cold Spring Harb. Symp. Quant. Biol..

[42] Moons P., Van Houdt R., Aertsen A., Vanoirbeek K., Engelborghs Y., Michiels C.W. (2006). Role of quorum sensing and antimicrobial component production by *Serratia. plymuthica* in formation of biofilms, including mixed biofilms with *Escherichia coli*. Appl. Environ. Microbiol..

[43] Aertsen A., Faster D., Michiels C.W. (2005). Induction of Shiga toxin-converting prophage in *Escherichia coli* by high hydrostatic pressure. Appl. Environ. Microbiol..

[44] Dunn J.J., Studier F.W. (1983). Complete nucleotide sequence of bacteriophage T7 DNA and the locations of T7 genetic elements. J. Mol. Biol..

[45] Christensen B.B., Sternberg C., Andersen J.B., Palmer R.J., Nielsen A.T., Givskov M., Molin S. (1999). Molecular tools for study of biofilm physiology. Meth. Enzymol..

[46] Fogg P.C., Saunders J.R., McCarthy A.J., Allison H.E. (2012). Cumulative effect of prophage burden on Shiga toxin production in *Escherichia coli*. Microbiology.

[47] James C.E., Stanley K.N., Allison H.E., Flint H.J., Stewart C.S., Sharp R.J., Saunders J.R., McCarthy A.J. (2001). Lytic and lysogenic infection of diverse *Escherichia coli* and *Shigella.* strains with a verocytotoxigenic bacteriophage. Appl. Environ. Microbiol..

[48] Gamage S.D., Strasser J.E., Chalk C.L., Weiss A.A. (2003). Nonpathogenic *Escherichia coli* can contribute to the production of Shiga toxin. Infect. Immun..

[49] Tóth I., Schmidt H., Dow M., Malik A., Oswald E., Nagy B. (2003). Transduction of porcine enteropathogenic *Escherichia coli* with a derivative of a shiga toxin 2-encoding bacteriophage in a porcine ligatedileal loop system. Appl. Environ. Microbiol..

[50] Cornick N.A., Helgerson A.F., Mai V., Ritchie J.M., Acheson D.W. (2006). *In vivo* transduction of anStx-encoding phage in ruminants. Appl. Environ. Microbiol..

[51] Hastings P.J., Rosenberg S.M., Slack A. (2004). Antibiotic-induced lateral transfer of antibiotic resistance. Trends Microbiol..

[52] Zhang X., McDaniel A.D., Wolf L.E., Keusch G.T., Waldor M.K., Acheson D.W. (2000). Quinolone antibiotics induce Shiga toxin-encoding bacteriophages, toxin production, and death in mice. J. Infect. Dis..

[53] Aertsen A., Van Houdt R., Michiels C.W. (2005). Construction and use of an *stx1* transcriptional fusion to *gfp*. FEMS Microbiol. Lett..

[54] Carson L., Gorman S.P., Gilmore B.F. (2010). The use of lyticbacteriophages in the prevention and eradication of biofilms of *Proteus mirabilis* and *Escherichia coli*. FEMS Immunol. Med. Microbiol..

[55] Chibeu A., Lingohr E.J., Masson L., Manges A., Harel J., Ackermann H.W., Kropinski A.M., Boerlin P. (2012). Bacteriophages with the ability to degrade uropathogenic *Escherichia coli* biofilms. Viruses.

[56] Corbin B.D., McLean R.J., Aron G.M. (2001). Bacteriophage T4 multiplication in a glucose-limited Escherichia coli biofilm. Can. J. Microbiol..

[57] Buckling A., Rainey P.B. (2002). Antagonistic coevolution between a bacterium and a bacteriophage. Proc. Biol. Sci..

[58] Kashiwagi A., Yomo T. (2011). Ongoing phenotypic and genomic changes in experimental coevolution of RNA bacteriophage Qβ and *Escherichia coli*. PLoSGenet..

[59] Lu T.K., Collins J.J. (2009). Engineered bacteriophage targeting gene networks as adjuvants for antibiotic therapy. Proc. Natl. Acad. Sci. USA.

[60] Ryan E.M., Alkawareek M.Y., Donnelly R.F., Gilmore B.F. (2012). Synergistic phage-antibiotic combinations for the control of *Escherichia coli* biofilms *in vitro*. FEMS Immunol. Med. Microbiol..

